# Effects of Red Mud on Cement Mortar Based on Sodium Salt Type

**DOI:** 10.3390/ma18153563

**Published:** 2025-07-30

**Authors:** Suk-Pyo Kang, Sang-Jin Kim, Byoung-Ky Lee, Hye-Ju Kang

**Affiliations:** 1 Department of Construction Engineering, Woosuk University, Jincheon 27841, Republic of Korea; ksp0404@woosuk.ac.kr; 2 Department of Architecture, Woosuk University, Jincheon 27841, Republic of Korea; 3 Industrial Tools Circulating Center, COCHEMS Co., Ltd., 160, Daehwa-ro, Daedeok-gu, Daejeon 34368, Republic of Korea; fluolbk@naver.com; 4 Construction Test & Assessment Center, Construction Test & Certification Department, Korea Institute of Civil Engineering and Building Technology, Goyang-si 10223, Republic of Korea; kanghyeju@kict.re.kr

**Keywords:** recycling, red mud, liquefied red mud, acid treatment, cement mortars

## Abstract

This study treated the NaOH component in red mud sludge, an industrial by-product generated at 300,000 tons annually in Korea, with sulfuric and nitric acids to produce NaSO_4_ and NaNO_3_, respectively. The effects of acid-treated liquid red mud (LRM) on the hydration reactions and early strength development in cement mortar were investigated. Properties such as flow, setting time, hydration heat, and compressive strength were evaluated alongside hydration product analysis using X-ray diffraction (XRD) and scanning electron microscopy (SEM). The neutralization of LRM stabilized the pH between 7 and 8. Mortars containing neutralized red mud (NRM) and sulfuric-treated red mud (SRM) exhibited shorter initial setting times and similar final setting times compared to untreated red mud (LM). After one day, XRD confirmed the presence of Ca(OH)_2_ in NRM and SRM but not in LM, while SEM revealed reduced pore sizes in NRM and SRM. Depending on dosage, the compressive strength of SRM increased by 35–60% compared to Plain mortar. These results demonstrate that LRM treated with nitric or sulfuric acid has significant potential as a setting accelerator for cement mortar.

## 1. Introduction

With the increasing amount of waste produced by industrial development and mass production, waste treatment has become a severe environmental issue [1]. The development of recycling technologies that substitute waste for natural resources may play a vital role in overcoming modern waste management issues. [2].

Red mud is generated as strongly alkaline (pH: 10–12.8) sludge during the Bayer process of producing alumina as it contains the sediment of solid compounds and residual sodium hydroxide solutions. The Bayer process, discovered by the Australian chemist K. J. Bayer in 1887, extracts aluminum hydroxide from bauxite [3]. In this process, bauxite is dissolved in a sodium hydroxide solution to settle insoluble impurities (e.g., iron, silicon, and titanium) as solid compounds and elute only aluminum compounds. Subsequently, seeds are added to the oversaturated sodium aluminate solution to precipitate aluminum hydroxide [4].

The production of one ton of alumina results in the generation of about 1.5 tons of red mud, contributing to an annual global output of nearly 150 million tons. When landfilled for disposal, red mud, which is highly alkaline (pH: 11–12), causes soil, water, and air pollution, a significant concern in industries [5]. With the growth of the aluminum industry and the increase in the number of factories, the amount of red mud generated worldwide is estimated to be 660 million tons per year [6,7]. Enormous efforts have been adopted for treating and recycling red mud; however, an economical and practical technology has not yet been developed [8].

In Korea, red mud sludge is generated at an annual rate of 300,000 tons [9] and is designated as recyclable general waste under the Wastes Control Act. It can be recycled into non-metal mineral products, such as aggregates, glass, cement, concrete, ready-mixed concrete, refractories, ceramics, and various stone products. It can also be used in asphalt concrete, asphalt, binders, landfill cover soil, marine landfill cover soil, and liner and cover materials, provided that the content of harmful substances remains below the regulatory thresholds. Recently, red mud has also been applied to room-temperature asphalt mixtures in combination with waste glass [10].

Currently, research on bauxite residue reutilization primarily focuses on the following aspects: recovery of value metals (e.g., iron, aluminum, titanium, gallium, rhenium and rare earth elements); production of construction materials such as cement, concrete, bricks, geopolymers, ceramic materials, road base and subgrade materials, water-resistant particleboards combined with wood waste, etc.; preparation of remediation materials applied to managing the polluted gas, water, and soil; stabilization/solidification of As-contaminated marine sediment; application as a catalyst for various reactions; and other aspects [11].

However, the strong alkalinity limits the utilization of bauxite residue in these fields. None of these applications has yet been commercially implemented on an industrial scale [12]. For instance, high alkalinity can cause alkali efflorescence during the production of construction materials, leading to low strength and poor durability [9]. Additionally, it increases acid consumption and cost during valuable metals leaching [13]. Therefore, dealkalization of bauxite residue is necessary before following disposal or comprehensive utilization.

Theoretically, since bauxite residue primarily contains soluble and structural alkalis, the principle for its dealkalization can be divided into two aspects: (1) converting insoluble alkalis into soluble ones by breaking down their structure and subsequently removing them via water washing; (2) converting soluble alkalis into insoluble ones through precipitation, thereby suppressing their dissolution [14]. However, in practice, these two principles are often combined to achieve the dealkalization of bauxite residue. The reported management of bauxite residue alkali has subsequently been comprehensively reviewed based on four categories: acid neutralization, salt (ion) precipitation or displacement, metallurgy, and microbial-driven remediation.

Acid neutralization is a simple and efficient method for removing alkali from bauxite residues, typically using sulfuric and nitric acid. When feasible, the waste acid generated from other industrial processes offers a more economical option. Most studies on dealkalization by acid neutralization focus on the safe disposal and comprehensive utilization of red mud. However, there is relatively limited research on recycling acid-neutralized red mud in the construction industry. In particular, studies on the effect of the alkali salt generated by acid neutralization on cement hydration reactions and initial strength characteristics are limited. Sodium salts, such as sodium sulfate and sodium nitrate, formed by neutralizing sodium with acid, are considered hydration accelerators for cement mortar and concrete since they can accelerate the hydration rate of cement and improve compressive strength at early ages depending on their content [15]. Recycling dealkalized red mud as a cementitious material contributes to ecological environmental protection and presents new directions for low-carbon cement/concrete development [16].

In this study, the NaOH component abundant in the red mud sludge generated in Korea was treated with sulfuric acid and nitric acid to convert it into sodium sulfate and sodium nitrate. The properties and hydration products of cement mortar were analyzed to intensively examine the effects of sodium salt at early ages, as presented in the existing literature.

## 2. Materials and Methods

### 2.1. Materials

Table 1 presents the chemical composition of the red mud sludge sourced from KC in Mokpo City, Republic of Korea. This sludge exhibited a moisture content of 36 wt% and a corresponding solid fraction of 64 wt%. Upon drying, the solid phase was examined via X-ray fluorescence (XRF), revealing that SiO_2_, Al_2_O_3_, Fe_2_O_3_, and Na_2_O made up roughly 90 wt% of the total composition.

For chemical modification of the liquid red mud, sulfuric acid (95% purity, Daejung Chemicals & Metals Co., Ltd., Siheung-si, Republic of Korea) and nitric acid (60% concentration, Samchun Pure Chemical Co., Ltd., Pyeongtaek, Republic of Korea) were utilized. Ordinary Portland cement (OPC), procured from Hanil Cement Co., Ltd. (Seoul, Republic of Korea), served as the binder for mortar fabrication, with its physicochemical characteristics detailed in Table 2. As fine aggregate, natural sand composed predominantly of SiO_2_ (≥98%) and featuring rounded particles up to 1.6 mm in diameter was used.

The properties of the dispersant (polycarboxylic-acid-based, from Dow Inc., Midland, MI, USAS) and thickener (methylcellulose-based, from Kao Corporation, Tokyo, Japan), both employed in the preparation of liquefied red mud (LRM), are shown in Table 3. A transparent polyoxyalkylene-alkylether-based agent, supplied by PCC Exol SA, Brzeg Dolny, Poland, was used as the defoaming agent, with a pH range of 4.5–7.5, a specific gravity of 0.9984, and a viscosity of 269 cP at 25 °C.

### 2.2. Methods

#### 2.2.1. Sample Preparation

**Figure 1:**
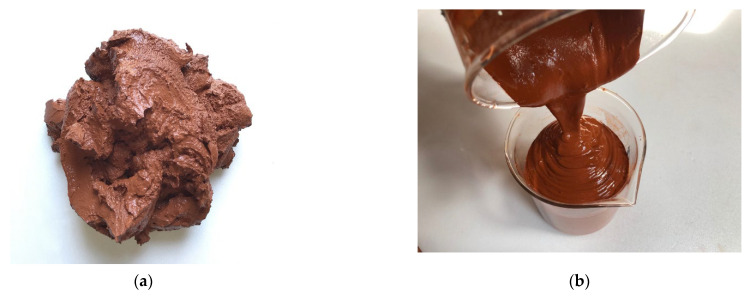
Red Mud sludge and liquefied red mud. (**a**) Red Mud sludge. (**b**) Liquefied Red Mud.

Liquefied red mud (LRM) was produced by blending red mud sludge with water, a dispersing agent, and a defoamer at a mass ratio of 1:0.2:0.0036:0.0014, respectively. Initially, the sludge and water were mixed at 20,000 rpm for 3 min using a Homo Mixer (K & S Co., Mokpo, Republic of Korea), as illustrated in Figure 1 [17]. Following this step, the dispersant and defoaming agent were introduced based on the specified proportions and the mixture was further stirred for an additional 2 min.

Subsequent analyses were conducted to determine the physical and chemical characteristics of the resulting LRM. The pH level of the LRM, adjusted using nitric acid and sulfuric acid, was controlled within the range of 7 to 8. Mineralogical analysis of the samples—LRM, LRM neutralized with nitric acid (LRM+N), and with sulfuric acid (LRM+S)—was carried out using X-ray diffraction (XRD) techniques.

Table 4 outlines the mixture design of the cement mortars incorporating LRM, LRM+N, and LRM+S as partial cement replacements. The W/C ratio of Plain was set to 50%. Additionally, the W/C ratios of LM, NRM, and SRM were set to 50%. The amount of mixing water was determined based on the moisture content of LRM. The cement mortar was mixed for 4 min using a mortar mixer (Heungjin, Republic of Korea). Cement mortars that contained 5 wt.% of LRM were referred to as LM5 and LM10. Cement mortars that contained 5 wt.% of LRM+N were referred to as NRM5 and NRM10. Cement mortars that contained 5 wt.% of LRM+S were named SRM5 and SRM10.

#### 2.2.2. Testing Methods

The flowability of the hydraulic cement mortar was examined following the ASTM C1437 standard [18], where the mortar’s spread was measured at four equidistant points, and the average diameter was used to assess its workability. The setting behavior of the cement was identified through the Vicat needle method as described in ASTM C191 [19]. For hydration analysis, a TAM AIR microcalorimeter (C80, SETARAM, Plan-les-Ouates, Switzerland) equipped with multiple channels was employed to monitor heat flow over a 72 h period immediately after combining cement with water containing either liquefied red mud (LRM) or its nitric acid-neutralized form (LRM+N) [20]. Compressive strength testing conformed to ASTM C349 [21], using a universal testing machine (Heungjin Testing Machine Co., Ltd., Gimpo-si, Republic of Korea). Mortar specimens, measuring 40 × 40 × 160 mm^3^, were cured under conditions of 20 ± 2 °C and 50% relative humidity, and strength was evaluated at 1, 3, 7, and 28 days based on the average of three samples. For hydration product analysis, samples cured for 1 and 28 days were immersed in anhydrous ethanol to stop further hydration, then dried at 40 °C for 24 h. These samples were ground, passed through a 200-mesh sieve, and examined by X-ray diffraction (XRD, SmartLab, Rigaku, Tokyo, Japan) using Cu-Kα radiation at 45 kV, 200 mA, and a scan rate of 4°/min within the 2θ range of 5–75° [22]. Microstructural features were also observed using field emission scanning electron microscopy (FE-SEM, S-4800, Hitachi, Tokyo, Japan) after applying the same ethanol immersion and drying process to the samples at 1 and 28 days of curing.

## 3. Results and Discussion

### 3.1. Properties of LRM+N and LRM+S

#### 3.1.1. XRD

**Figure 2:**
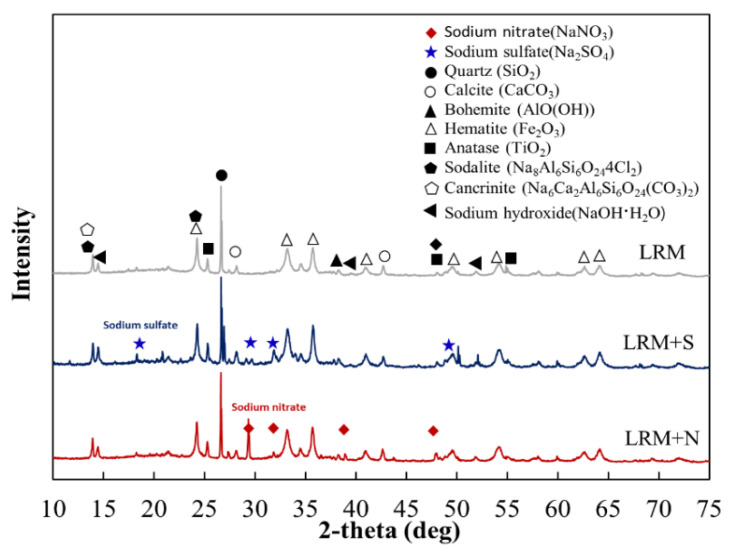
XRD patterns of LRM, LRM+N, and LRM+S.

The mineral composition of the LRM neutralized by nitric acid and sulfuric acid was analyzed. The results shown in Figure 2 indicate that the primary compounds of LRM were found to be quartz, calcite, bohemite, and hematite [23]. These compounds were also found in LRM+S and LRM+N. For LRM+S, the characteristic peaks were observed at 2θ = 18.1, 29.6, 31.9, and 49.5°, corresponding to Na_2_SO_4_, due to the addition of sulfuric acid. In the case of LRM+N, the characteristic peaks were identified at 2θ = 29.4, 31.8, 38.9, and 47.9°, corresponding to NaNO_3_, due to the addition of nitric acid.

#### 3.1.2. Physical Properties

Table 5 shows the physical properties of LRM, LRM+S, and LRM+N.

After LRM was treated with nitric acid and sulfuric acid, the pH was stabilized as it was maintained at 11.5, 7.5, and 7.6, respectively, for seven days. The viscosity of the sample modified with nitric acid and sulfuric acid increased compared to LRM. In particular, the viscosity of LRM+S treated with sulfuric acid increased to 60,670 cP.

### 3.2. Flow

**Figure 3:**
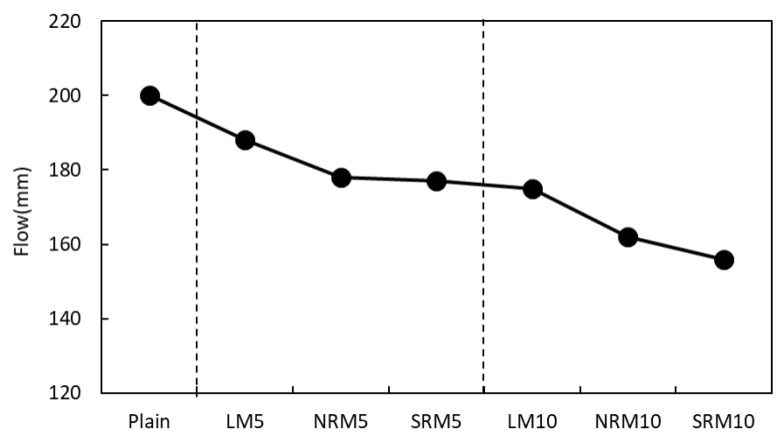
Flow of cement mortars with varying proportions of LRM, LRM+N, and LRM+S.

The flow of cement mortar that contained LRM was compared with that of Plain, as shown in Figure 3. The flow of LM was lower than that of Plain. As the content of LRM increased, the flow value decreased. In particular, the flow of NRM and SRM, which are samples that used LRM after modification, was lower than that of the LM samples; this is likely because the viscosity of the LRM neutralized by nitric acid and sulfuric acid increased.

### 3.3. Setting Time

**Figure 4:**
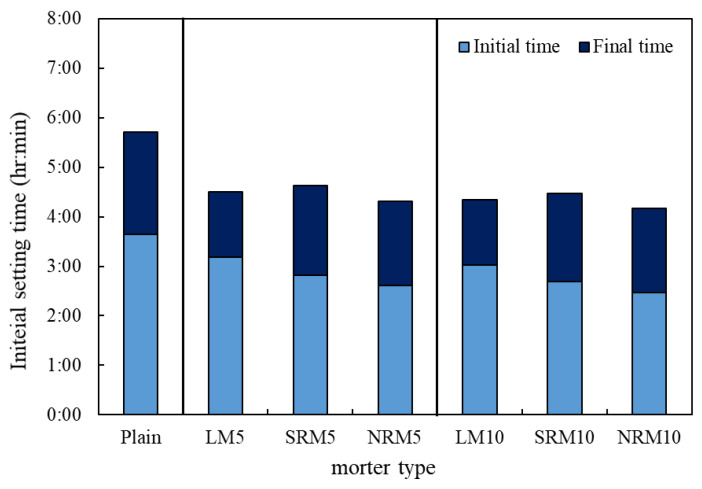
Initial and final setting times of cement mortars with varying proportions of LRM, LRM+N, and LRM+S.

The setting time of cement mortar containing LRM was compared with that of Plain, as shown in Figure 4. The initial and final setting times of LM were shorter than those of Plain. For LM5 and LM10, the initial setting times were reduced by 28 and 37 min, and their final setting times were reduced by 73 and 82 min, respectively, compared to Plain; this indicates that using LRM had a greater impact on the final setting time than the initial setting time due to the NaOH component effect [24].

Meanwhile, the final setting times of NRM and SRM were not significantly different from that of LM, but their initial setting times were slightly shorter (refer to the influence of Na_2_SO_4_ and NaNO_3_).

Therefore, neutralizing LRM and incorporating it into the cement mortar reduced the initial setting time.

### 3.4. Hydration Heat

**Figure 5:**
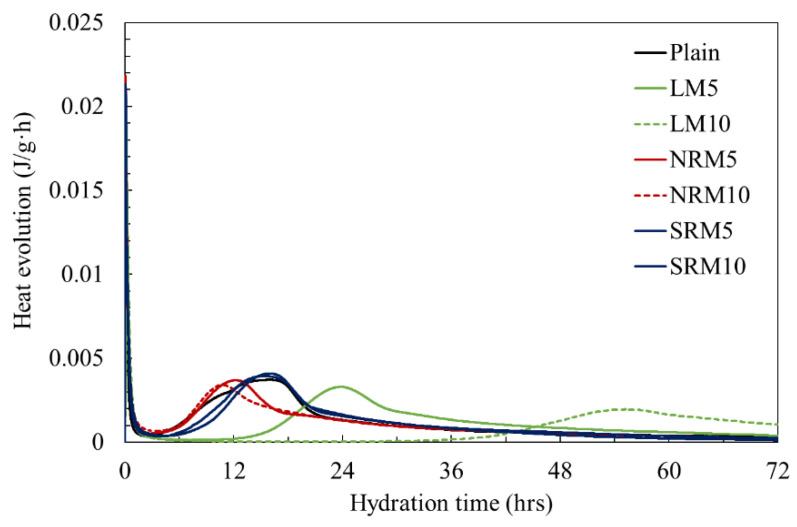
Heat evolution rate of cement pastes with red mud.

Figure 5 shows the evolution of hydration heat over time. Typically, the evolution of hydration heat in cement mortar is influenced by the hydration reactions of tricalcium silicate (C_3_S) and dicalcium silicate (C_2_S), which lead to the formation of calcium silicate hydrate (C-S-H) and portlandite [25]. In the case of the plain sample, the heat release peaked around 15 h. However, for the unmodified

Liquefied red mud (LM), the maximum heat flow was observed much later—between 24 and 54 h—indicating a delay ranging from approximately 1.6 to 3.6 times that of the plain sample. In contrast, when LRM was chemically treated with nitric acid (NRM) or sulfuric acid (SRM), the peak hydration times closely resembled those of the plain mortar, irrespective of the quantity incorporated. These findings imply that, when pre-treated with acidic modifiers, LRM participates in the hydration process in a manner similar to the plain sample, particularly in terms of C_3_S and C_2_S reactions and the subsequent generation of C-S-H and portlandite.

**Figure 6:**
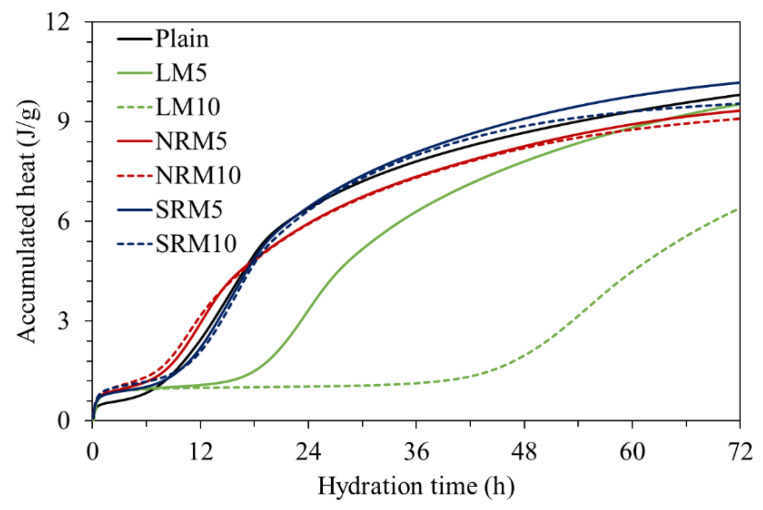
Accumulative heat of cement pastes with red mud.

Figure 6 demonstrates the cumulative hydration heat over time. Over a 72 h period, the total hydration heat released by the plain sample reached 9.8 J/g. In comparison, the unmodified liquefied red mud (LM) resulted in cumulative values ranging from 6.4 to 9.5 J/g. Upon modification with nitric acid, the LRM exhibited a hydration heat between 9.1 and 9.5 J/g, while sulfuric acid treatment led to slightly higher values, ranging from 9.5 to 10 J/g. These results suggest that pretreating LRM with acidic agents such as nitric or sulfuric acid prior to incorporation into cement mortar can enhance early-age strength development. Moreover, the variation in the amount of modified LRM used appears to exert only a marginal influence on the hydration heat outcome.

### 3.5. Compressive Strength

**Figure 7:**
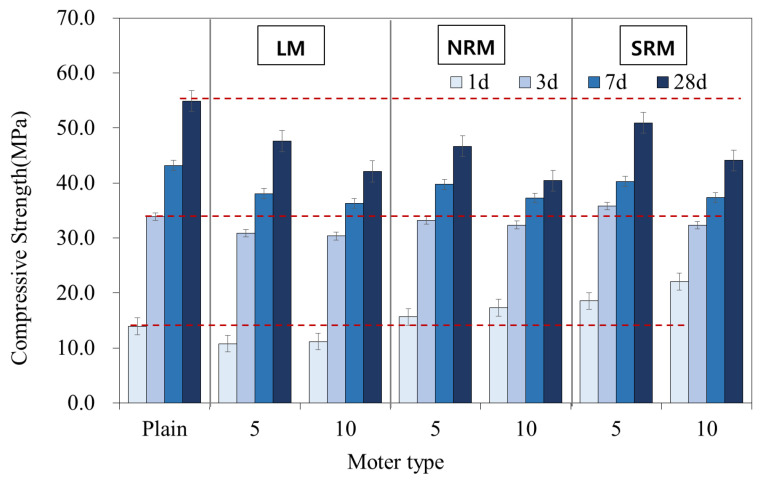
Effects of LRM and LRM+N on the compressive strength of cement mortar. The red dotted lines are used as reference lines to visually compare the compressive strength values of the different samples with the plain sample.

Figure 7 shows the compressive strength of cement mortar that used LRM according to the modification method.

At 28 days of age, the compressive strengths were 54.91 MPa for Plain, 42.1–47.6 MPa for LM, 40.4–46.7 MPa for NRM, and 44.1–50.9 MPa for SRM. The compressive strength of LM was lower than that of Plain. The compressive strength of NRM was similar to that of LM, but the strength of SRM was improved compared to LM. In addition, the compressive strength at 28 days of age decreased as the contents of LRM, LRM+N, and LRM+S increased.

**Figure 8:**
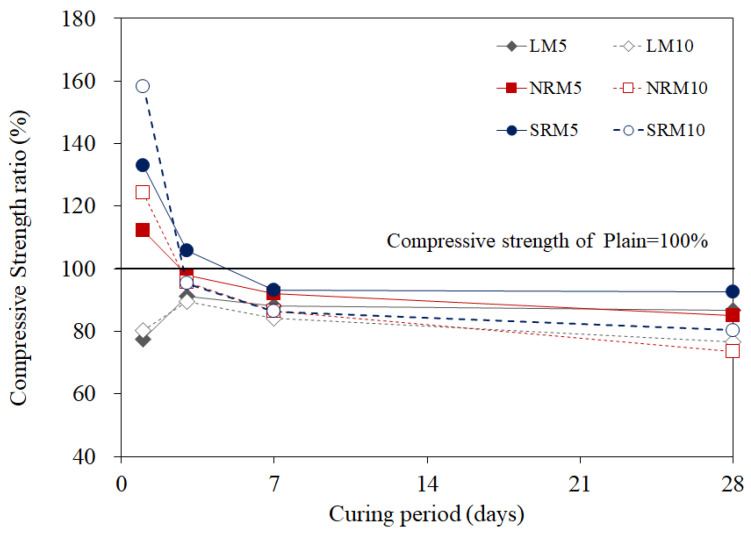
Compressive strength ratios of LRM and LRM+N-modified cement mortar over the aging period.

Figure 8 shows the compressive strength ratios between Plain and cement mortar that used LRM according to the modification method.

The compressive strength of LM was significantly lower compared to Plain, with a reduction of more than 20% at one day of age. It is reported that adding an appropriate amount of NaOH to OPC improves initial strength, but adding an excessive amount decreases the strength [26]. The substantial reduction in initial strength is likely due to its high NaOH content.

However, the compressive strengths of NRM and SRM were generally higher than that of LM. In particular, the compressive strengths of NRM and SRM at one day of age increased by 112–124% and 133–158% compared to Plain, respectively. The one-day compressive strength of SRM was approximately double that of LM.

### 3.6. XRD Observations

**Figure 9:**
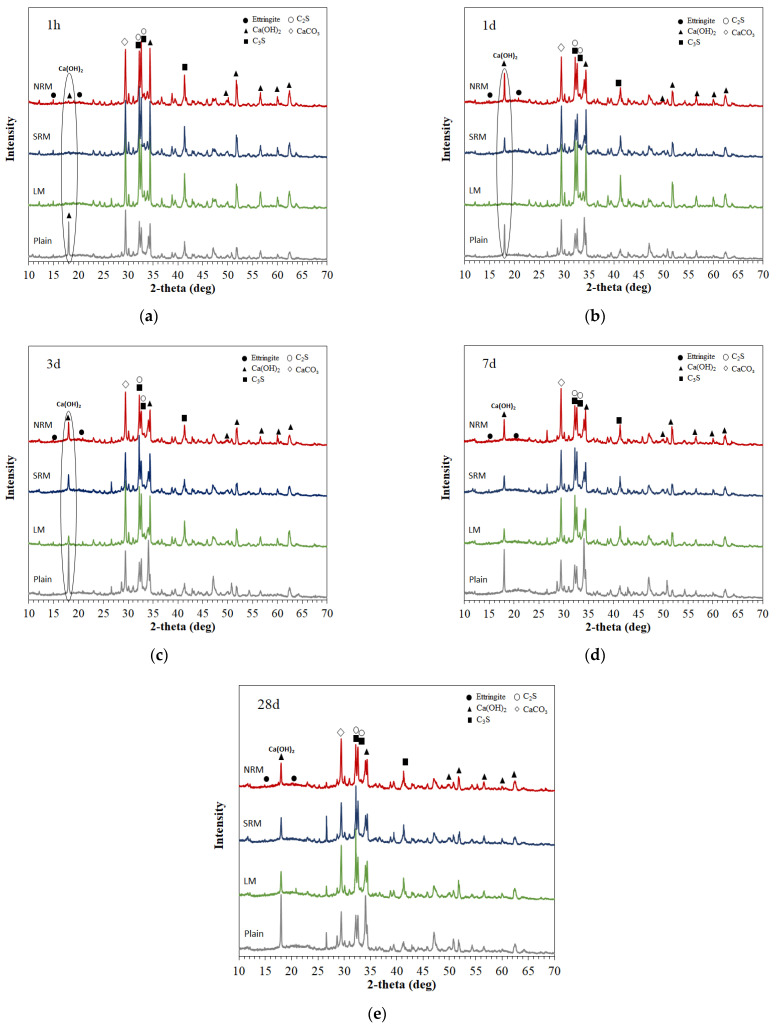
XRD results of Plain, LM10, NRM10, and SRM10 mortars after (**a**) 1 h and (**b**) 1 day. (**c**) 3 days (**d**) 7 days (**e**) 28 days.

Figure 9 illustrates the XRD analysis results of cement mortar that used LRM according to the modification method by age.

The peaks of Ca(OH)_2_, which is the hydration product of Plain, were identified at 2θ = 18, 34, 51, 53, 57, and 60° after 1 h. For unmodified LM, however, the Ca(OH)_2_ peaks were identified after three days. It was reported that adding NaOH to cement hydration reactions interferes with normal hydration reactions because the increased pH value increases the Si concentration and decreases the Ca concentration in the solution [27]. Consequently, the amount of Ca(OH)_2_ generated decreases as the NaOH concentration increases [28]. It is judged that the Ca(OH)_2_ peaks of unmodified LM containing a significant amount of NaOH indicate that normal hydration reactions were not performed until three days of age, and, thus, a significantly low compressive strength was developed compared to Plain. Conversely, the Ca(OH)_2_ peaks of NRM and SRM, which modified LRM with nitric acid and sulfuric acid, were identified after one day, indicating that normal hydration reactions were performed, and the compressive strength was improved compared to LM.

### 3.7. SEM Observations

**Figure 10:**
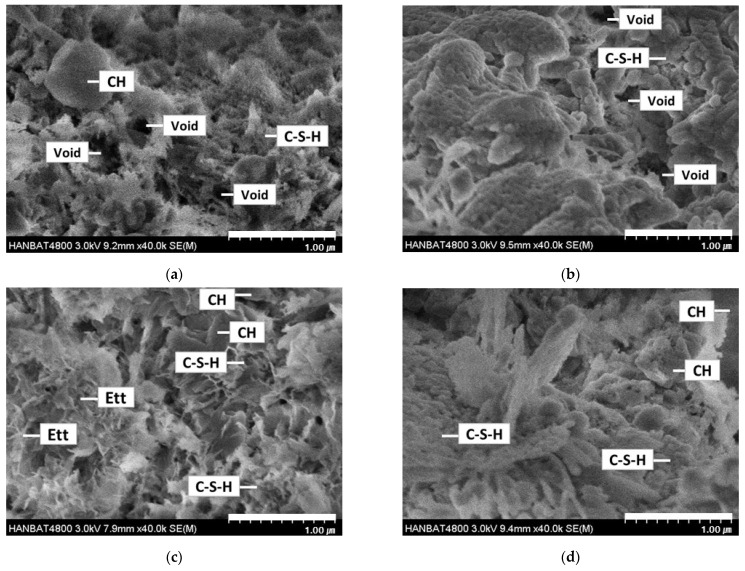
SEM images of Plain, LM10, SRM10, and NRM10 mortars at 1 day of age: (**a**) Plain, (**b**) LM10, (**c**) SRM10, and (**d**) NRM10.

Figure 10 shows the microstructure images of cement mortar that used LRM according to the modification method at one day of age.

Voids, CH, and C-S-H were observed in the microstructure of Plain. However, in the hydration products of unmodified LM, several voids and differently shaped C-S-H were observed, with CH notably absent, indicating different hydration reactions from Plain. This observation was consistent with the XRD results.

For the hydration products of NRM and SRM, which involved LRM modified with nitric acid and sulfuric acid, voids were reduced, and CH was observed compared to LM. In particular, ettringite generation was observed in SRM; this seems to have affected an increase in the initial strength of SRM. LRM+S used in SRM modified LRM with sulfuric acid, thereby reducing NaOH and generating Na_2_SO_4_, as illustrated in Figure 2. The existing literature reports that adding Na_2_SO_4_ improves the initial strength by filling voids and reducing the porosity of the entire capillary by generating ettringite at the beginning of cement hydration [29]. The shapes of hydration products were planar or foil-like shapes in LM10; however, they changed into radiating needle shapes in SRM10. They were similar to the shapes of the cement hydration reaction products obtained by adding NaOH and Na_2_SO_4_ to C_3_S cement paste [15].

## 4. Conclusions

This study converted the NaOH component abundant in the red mud sludge generated in Korea into sodium sulfate and sodium nitrate by treating it with sulfuric acid and nitric acid, respectively. An analysis was conducted to evaluate how these sodium salts affect the early-stage behavior and resulting hydration phases of cement mortar.

(1) LRM was neutralized with nitric acid and sulfuric acid, confirming the production of NaNO_3_ and Na_2_SO_4_ and stabilizing the pH between 7 and 8.
(2) NRM and SRM, with the addition of LRM+N and LRM+S, had a shorter initial setting time and a similar final time compared to LM. The hydration heat peak of NRM and SRM appeared earlier than that of LM and was comparable to Plain.
(3) XRD analysis at 1 day revealed that the Ca(OH)_2_ peak in NRM and SRM was similar to Plain but absent in LM. SEM observations result at 1 day showed reduced pore structures and the presence of Ca(OH)_2_ in NRM and SRM compared to LM.
(4) The 1-day compressive strength of NRM and SRM exceeded that of LM, with SRM demonstrating a 35–60% strength increase over Plain, depending on dosage. These findings suggest that LRM treated with nitric or sulfuric acid has potential as a setting accelerator for cement mortar.

Future research should explore the long-term durability and mechanical properties of mortar containing LRM+N and LRM+S, investigate their performance under various environmental conditions, and evaluate their economic and environmental benefits for broader industrial applications. Additionally, understanding the hydration mechanisms in greater depth and testing the applicability of this approach to different red mud sources will be critical for expanding its practical use.

## Figures and Tables

**Table 1:** Chemical composition of red mud sludge.

Type	Chemical Composition (wt.%)											Moisture Content Ratio (wt.%)
	SiO_2_	Al_2_O_3_	Fe_2_O_3_	CaO	MgO	SO_3_	Na_2_O	K_2_O	TiO_2_	MnO	Cr_2_O_3_	
Red mud sludge	38.8	16.1	22.8	3.4	0.21	0.29	10.0	0.4	6.2	0.1	0.1	36

**Table 2:** Physical properties and chemical composition of OPC.

Type	Blaine (cm^2^/g)	Setting Time	Density (g/cm^3^)	Chemical Composition (wt.%)							
		Initial (min)	Final (h)		SiO_2_	Al_2_O_3_	Fe_2_O_3_	CaO	MgO	SO_3_	Lg. Loss
OPC *	3300	200	5.5	3.15	21.7	5.7	3.2	63.1	2.8	2.2	1.3

* OPC: ordinary Portland cement.

**Table 3:** Properties of admixtures.

Type	Color	Specific Gravity	Residue Content (%)	Viscosity (cP)	NaCl (wt.%)	Moisture (wt.%)
Polycarboxylic acid series	Light brown	1.136	40.7	180	-	-
Methyl cellulose	White	-	-	32,900	1.36	1.40

**Table 4:** Mix design of cement mortar.

Mix ID	W/C (%)	Mix Design (g)					
		Cement	Sand	Water	LRM	LRM+N	LRM+S
Plain	50	100	300	50	-	-	-
LM5 *		95		45.27	9.72	-	-
LM10		90		40.54	19.46	-	-
NRM5 **		95		45.51	-	9.49	-
NRM10		90		41.02	-	18.98	-
SRM5		95		46.06		-	8.94
SRM10		90		42.12		-	17.88

* Liquefied red mud + mortar. ** Nitric acid neutralization liquid red mud + mortar.

**Table 5:** Physical properties of LRM.

Type of Red Mud	Moisture Content (%)	pH	Density (g/cm^3^)	Viscosity (cP)
LRM	48.6	11.5	1.50	36,670
LRM+N	47.3	7.5	1.50	43,650
LRM+S	44.1	7.6	1.54	60,670

## Data Availability

The data presented in this study are available on request from the corresponding author. The data are not publicly available due to confidentiality agreements with the research sponsors.

## Abbreviations

The following abbreviations are used in this manuscript:

LRM	Liquefied red mud
NRM	Neutralized red mud
OPC	Ordinary Portland cement
SRM	Sulfuric-treated red mud
XRD	X-ray diffraction
SEM	Scanning electron microscopy
